# Orchestrating the Selection and Packaging of Genomic RNA by Retroviruses: An Ensemble of Viral and Host Factors

**DOI:** 10.3390/v8090257

**Published:** 2016-09-20

**Authors:** Rebecca J. Kaddis Maldonado, Leslie J. Parent

**Affiliations:** 1 Division of Infectious Diseases and Epidemiology, Department of Medicine, Penn State College of Medicine, 500 University Drive, Hershey PA 17033, USA; rjk297@psu.edu; 2 Department of Microbiology and Immunology, Penn State College of Medicine, 500 University Drive, Hershey PA 17033, USA

**Keywords:** retroviruses, viral RNA packaging, retroviral Gag proteins, subcellular trafficking, virus–cell interactions, viral RNA export, retrovirus assembly

## Abstract

Infectious retrovirus particles contain two copies of unspliced viral RNA that serve as the viral genome. Unspliced retroviral RNA is transcribed in the nucleus by the host RNA polymerase II and has three potential fates: (1) it can be spliced into subgenomic messenger RNAs (mRNAs) for the translation of viral proteins; or it can remain unspliced to serve as either (2) the mRNA for the translation of Gag and Gag–Pol; or (3) the genomic RNA (gRNA) that is packaged into virions. The Gag structural protein recognizes and binds the unspliced viral RNA to select it as a genome, which is selected in preference to spliced viral RNAs and cellular RNAs. In this review, we summarize the current state of understanding about how retroviral packaging is orchestrated within the cell and explore potential new mechanisms based on recent discoveries in the field. We discuss the *cis*-acting elements in the unspliced viral RNA and the properties of the Gag protein that are required for their interaction. In addition, we discuss the role of host factors in influencing the fate of the newly transcribed viral RNA, current models for how retroviruses distinguish unspliced viral mRNA from viral genomic RNA, and the possible subcellular sites of genomic RNA dimerization and selection by Gag. Although this review centers primarily on the wealth of data available for the alpharetrovirus Rous sarcoma virus, in which a discrete RNA packaging sequence has been identified, we have also summarized the *cis*- and *trans*-acting factors as well as the mechanisms governing gRNA packaging of other retroviruses for comparison.

## 1. Introduction

Retroviruses are positive-sense, single-stranded RNA viruses that are ubiquitous in nature, causing cancers and immunodeficiency syndromes in a variety of organisms, including humans. Retroviruses encode enzymes that reverse transcribe their viral RNA genomes (gRNA) into a double-stranded cDNA that integrates into the host-cell chromosome, resulting in the formation of a provirus. The integrated provirus serves as a template for transcription of the viral RNA (vRNA) by the host RNA polymerase II. Following transcription, the unspliced vRNA has three potential fates. The vRNA can be spliced into subgenomic messenger RNAs (mRNAs) that are exported from the nucleus via cellular transport pathways, and these mRNAs encode viral proteins such as the envelope glycoprotein. Alternatively, the vRNA can remain unspliced, and this full-length RNA serves two roles: (i) as mRNA for the translation of the retroviral proteins Gag and Gag–Pol; or (ii) as the gRNA that is packaged into new virions as a weak noncovalent dimer which undergoes further stabilization during virion maturation.

Encapsidation of gRNA is initiated when the retroviral Gag polyprotein binds to the highly-structured psi packaging sequence (known as Ψ) located in the 5′ untranslated region (UTR) of the vRNA. However, the mechanisms by which Gag identifies and selects the unspliced vRNA as its genome is unclear, and several major questions pertaining to gRNA packaging remain unanswered. For example, because full-length vRNA can be used either as mRNA or gRNA, is there a mechanism whereby Gag distinguishes between vRNA used for translation versus gRNA for encapsidation? Does the composition of the RNA binding proteins that interact with the unspliced vRNA co-transcriptionally in the nucleus define the cytoplasmic fate of the RNA [1,2,3]? If so, then which elements in the vRNA are required for the virus to sort the unspliced vRNA for various purposes?

Another puzzling topic in retroviral genome packaging pertains to the cellular location of the initial Gag–gRNA interaction. Because unspliced retroviral RNA is 5′ capped and 3′ polyadenylated, it is indistinguishable from cellular mRNAs, which are hundreds to thousands of times more abundant within the cell [4], lowering the probability that Gag interaction with the vRNA occurs by chance. Therefore, how does the Gag protein locate the gRNA and where in the cell does selection of the gRNA occur? Historically, it was thought that the Gag protein interacted with the unspliced vRNA in the cytoplasm or at the plasma membrane [5]. However, it was not clear how the gRNA was transported to the site of Gag interaction. What cellular or viral factors are involved in gRNA transport to the assembly site? Furthermore, when Gag initially binds to the gRNA, is the RNA in the form of a monomer or dimer? Although these questions still plague the field, studies of the avian retrovirus Rous sarcoma virus (RSV) have brought us closer to solving a few of these mysteries.

## 2. Trafficking of the RSV Gag Polyprotein

**Figure 1:**
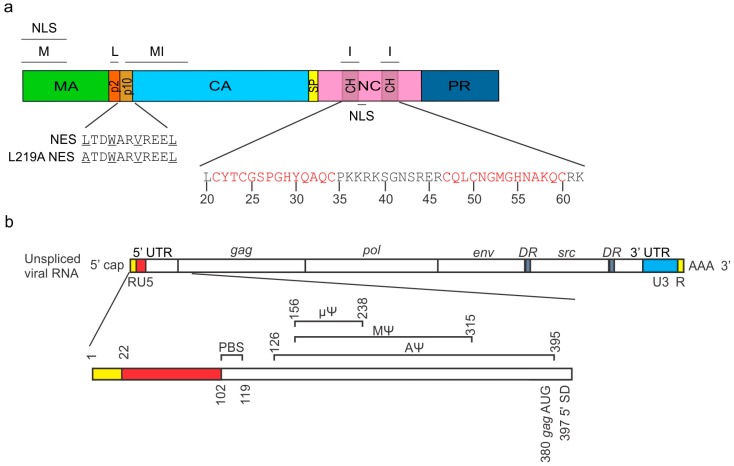
The Rous sarcoma virus (RSV) Gag polyprotein domain organization and functional sequences in the 5′ untranslated region (UTR) of the genomic RNA. (**a**) The RSV Gag protein is made of multiple domains: matrix (MA; green) contains the membrane binding motif (M) and a non-canonical nuclear localization signal (NLS); p2 (orange) contains the late (L) motif. p10 (gold) contains a chromosome region maintenance 1 (CRM1)-dependent nuclear export signal (NES); capsid (CA; blue) contains a multimerization interface (MI) that extends from p10; spacer peptide (SP; yellow); nucleocapsid (NC; pink) binds nucleic acids and proteins via interaction motifs (I) and Cys-His boxes (CH; translucent grey) and also contains an NLS; and protease (PR; blue). The Cys-His boxes contain zinc finger domains (red) required for the binding of the Ψ packaging sequence located in the 5′ UTR of the viral RNA (vRNA); (**b**) The primer binding site (PBS) is required for reverse transcription (nts 102–119). The entire RSV packaging sequence AΨ extends from nucleotides 126–395. The MΨ sequence extends from nucleotides 156–215. The minimal functional packaging element, identified as µΨ, is 82 nucleotides in length, extending from 156 to 238. Because the splice donor (SD) is located downstream of the packaging sequence at nucleotide 397, the Ψ sequence is contained in both spliced and unspliced vRNAs. DR: direct repeat; RU5: repeat-unique 5’; U3: unique 3’.

The RSV Gag polyprotein is translated in the cytoplasm from unspliced viral mRNA. Gag is comprised of multiple functional domains that are cleaved upon proteolytic cleavage by the protease domain (PR) during virion maturation (Figure 1). The domains of RSV Gag are matrix (MA), p2, p10, capsid (CA), spacer peptide (SP), nucleocapsid (NC), and PR. MA contains the plasma membrane targeting and binding motif (M) and a non-canonical nuclear localization signal (NLS) [6,7,8]. The p2 domain contains the late (L) motif and is involved in the pinching off of virions from the plasma membrane during budding [9,10,11]. The p10 domain contains a chromosome region maintenance 1 (CRM1)-dependent nuclear export signal (NES) and contains structural elements that influence mature virion morphology [6,12,13,14]. CA encompasses the major homology region (MHR) and the multimerization interface (MI), which facilitates Gag–Gag interactions in the immature and mature virus particle [5,14,15,16,17]. SP is required for the proper assembly of the immature virion [18]. NC contains two interaction (I) domains that mediate Gag–Gag and Gag–nucleic acid interactions, and NC contains both an NLS and a nucleolar localization signal [7,15,19,20].

## 3. Determinants of the RSV RNA Sequence Required for Packaging

### 3.1. The Psi Packaging Sequence

The initial step in particle assembly is the recognition, selection and binding of Gag to the gRNA for packaging into virions [5,21,22,23,24,25,26]. Encapsidation of the unspliced retroviral RNA is initiated by the binding of the NC domain of Gag to the highly structured Ψ packaging sequence located in the 5′ UTR [5,21,22,23,24,25,26,27,28,29,30] (Figure 1b). Although the RSV Ψ sequence is located on both the spliced and unspliced vRNAs, unspliced RNA is preferentially packaged at a ratio of approximately 200:1 unspliced to spliced vRNA [31]. In the absence of a Ψ-containing packageable vRNA, Gag will encapsidate a variety of cellular RNAs, including small noncoding RNAs such as 7SL, 5S ribosomal RNA (rRNA), microRNAs, primer and non-primer transfer RNAs (tRNAs), and U6 small nuclear RNA (snRNA), when forming virus-like particles [32,33,34,35,36,37,38,39,40,41,42,43,44,45,46,47].

The packaging sequence of RSV was discovered when spontaneously-arising mutant viruses were identified that were not infectious because they lacked the viral genome [48,49,50,51,52,53]. One such mutant, *SE*21Q1b, provided the foundation for the study of the role of the 5′ UTR in gRNA encapsidation [48,49,52]. The virus particles produced by this mutant are structurally similar to those of wild-type virus, except that they primarily contain cellular mRNAs, with less than 1% of the RNA being viral in origin [49,52]. Through restriction mapping and sequencing of the *SE*21Q1b provirus cloned from the c-*SE*21Q1b cell line, a 179-nucleotide deletion was mapped between positions 95 and 274 in the 5′ UTR, a region that includes the primer binding site (PBS), which is required for reverse transcription [48,54]. Another mutant identified in a different study (TK15), contained a 250-nucleotide deletion located between the PBS and the Gag initiation codon [50,51,53]. In addition to the RSV packaging mutants described above, mutational analysis of the 5′ long terminal repeat (LTR) of an avian sarcoma virus (ASV) provirus led to the discovery of several clones deficient in RNA packaging. These clones had various deletions in the 5′ UTR, although all of them had a common overlapping 30 base pair deletion that encompassed positions 218–248, which lie between the PBS (nucleotides 102–119) [5] and the Gag AUG (nucleotide 380) [55]. Moreover, in a gain-of-function experiment, an RNA containing the fusion of a 683 base pair region derived from the RSV 5′ LTR, encompassing 53 nucleotides of Unique 3’ (U3), all of Repeat-Unique 5’ (RU5), and the first 250 base pairs of *gag*, to a heterologous RNA coding for hygromycin resistance was packaged into virions when expressed in the Q2bn-4D cell line [56]. Together, these data indicate that this region in the 5′ UTR of the genome is both required for packaging gRNA and it is sufficient to induce packaging of a nonviral RNA sequence into virus particles.

The RSV packaging sequence has been characterized in a series of experiments that led to the identification of three regions of the 5′ UTR: AΨ (nucleotides 126–395) [57], MΨ (nucleotides 156–315) [31,58], and the minimal packaging sequence µΨ (nucleotides 156–238) [59] (Figure 1b). Each of these sequences is sufficient to allow the encapsidation of heterologous RNAs into virus particles produced from the Q2bn-4D packaging cell line, albeit at lower levels than the full-length RNA [60]. It is interesting that unspliced heterologous RNAs containing MΨ are packaged only about three-fold less efficiently than wild-type gRNA, even though viral *env* mRNAs are not efficiently packaged [31,57]. Furthermore, heterologous spliced RNAs containing AΨ are packaged at similar levels as unspliced AΨ-containing RNAs [31,57]. These findings are significant because the RSV Ψ sequence is present on both the spliced and unspliced vRNAs, yet the unspliced RNA is preferentially packaged into virions [31]. These data suggest that a secondary packaging signal exists within the RSV gRNA as a means to distinguish unspliced vRNA from spliced vRNA. Alternatively, long-range interactions of Ψ with downstream *cis*-acting elements may be needed to form the proper overall conformation needed for efficient gRNA packaging.

The RSV µΨ RNA element is highly structured, as determined by nuclear magnetic resonance (NMR) analysis [61,62]. Phylogenetic comparison of the 5′ UTRs of 13 strains of avian retroviruses revealed that the first 499 nucleotides of the leader contain several stem loops: leader loops (L1-5), the primer binding loop that encompasses the tRNA^Trp^ PBS, the O3 loop located just downstream of the upstream open reading frame 3 (uORF3) termination codon, and G1-3 loops located in the *gag* coding region. Furthermore, alignments of the 5′ UTR of these viruses with mutants deficient in gRNA packaging, mapped the Ψ sequence within the O3 stem loop (O3SL) [63,64]. Mutations that disrupt the O3 stem loop structure reduce gRNA packaging to less than 5% of wild-type levels [65]. However, mutations that maintain the structure of the main stem of the O3 loop decrease packaging to a lesser degree (30% of wild-type) [65]. The O3SL can be divided into three minor stem loops, O3Sla, b, and c. Changes within the minor loops that alter RNA structure greatly reduce packaging, whereas point mutations that maintain structure have no difference or only slightly decreased packaging levels compared to wild-type [58,59]. However, mutations in nucleotides upstream of O3Sla and O3Slb as well as those in the O3Slc loop that do not change the structure of the RNA also prevent binding of NC to µΨ in vitro and are no longer infectious [61,62]. These studies also revealed that the O3 stem loop alone (82 nucleotides long) is sufficient to allow the encapsidation of heterologous RNAs to the same level as MΨ [59], leading to the definition of the RSV minimal packaging sequence, µΨ. Together, these data suggest that both gRNA structure and sequence are important for efficient packaging, although structure of the O3 stem loop is the most important determinant of RSV gRNA encapsidation efficiency.

### 3.2. Mechanisms Governing Gag–vRNA Interaction

A major role of the Gag polyprotein is to select the unspliced vRNA as the genome to be packaged into nascently forming viral particles. The NC domain of Gag contains the major nucleic acid binding domain and is the contact point for interaction with Ψ. The nucleic acid binding activity of NC was discovered through electron micrographs showing that NC was bound to dimeric gRNA isolated from virions [66]. Furthermore, extraction and sedimentation of viral ribonucleoprotein complexes (RNPs) from virus particles revealed that NC associated with the vRNA has higher abundance compared to other components of Gag [67,68].

Alignments of the amino acid sequences of the RNA-binding motifs within Gag NC domains from multiple retroviruses, including ASV, revealed the presence of cysteine-histidine boxes (Cys-His) containing three cysteines and one histidine, which are conserved [69] (Figure 1a). The number of Cys-His boxes in NC vary among retroviruses (reviewed in [70]). Each of the Cys-His boxes of the RSV NC domain contain a zinc finger motif, CX_2_CX_4_HX_4_C, which coordinates Zn^2+^ [35] (Figure 1a). For RSV, the insertion or deletion of a single Cys-His box has negative effects on infectivity and gRNA packaging [71,72]. Insertion mutations that disrupt or lead to the loss of Cys-His boxes are non-infectious due to the inability of the virus to properly package dimeric gRNA. However, a small insertion added elsewhere in NC that does not disrupt the Cys-His boxes rendered a fully infectious virus [30]. Furthermore, deletion of either of the Cys-His boxes within NC leads to a decrease in genome packaging and loss of gRNA dimerization, whereas deletion of both Cys-His boxes completely abrogates gRNA encapsidation [72,73].

In addition to the Cys-His boxes being essential for encapsidation and dimerization of gRNA, the position of this motif and the amino acid identity of the surrounding residues are also essential [27,73]. For example, deletion of the proline between Cys24 and His29 leads to the loss of encapsidation, whereas changing the conserved Gly28 to Val was infectious but Gly28 to Ala was noninfectious, probably due to steric hindrance [27]. Furthermore, mutation of the basic residues directly downstream of the distal Cys-His box reduces packaging 25-fold [73]. Thus, the Cys-His boxes and the amino acid composition of the NC domain are essential for gRNA packaging and dimer formation in RSV.

To determine whether RSV Gag binds directly to the gRNA in an NC-dependent manner, a yeast three-hybrid study was performed in conjunction with RNA packaging assays [73,74,75,76]. If Gag bound to MΨ, then the activation domain fused to Gag would be brought into proximity of the DNA, inducing transcription of the *lacZ* reporter gene. In the presence of a wild-type Gag protein and MΨ, Gag bound to MΨ, resulting in β-galactosidase (β-gal) activity. However, upon deletion of the Cys-His boxes of NC, mutation of the basic residues downstream of the distal box, or use of an anti-sense MΨ, there was a decrease in β-gal activity [73]. Substitution of a single residue within the zinc fingers led to a 20–50 fold decrease of β-gal activity [75] and it was later shown that the zinc fingers of NC specifically bind to regions of the O3 stem loop in vitro [61]. Similarly, mutations in the O3 stem loop of MΨ that disrupted its structure also prevented Gag interaction, leading to loss of β-gal activity [76]. These results were supported by packaging studies in which the same mutants that failed to produce β-gal activity were also deficient in efficient gRNA packaging. In conclusion, these experiments illustrated the importance of the zinc fingers in Gag NC and the O3 stem loop of Ψ in vRNA for facilitating Gag–vRNA interactions.

Infectivity of a retroviral particle is dependent upon the encapsidation of a non-covalently-linked gRNA dimer (reviewed in [77]). Using Gag mutants containing a defective viral protease, it was found that NC packages gRNA in the context of the full-length Gag polyprotein, but the formation of a gRNA dimer depends upon Gag cleavage during particle maturation after budding [21,22]. These data suggest that dimerization of the RSV gRNA is promoted by NC after particle maturation. Analysis of rapid-harvest virions collected soon after release from an infected cell revealed that gRNA in those particles are primarily monomeric compared to mature virions that contain dimeric gRNA [23,25,78]. These data suggest that RSV gRNA is packaged as a monomer and that dimer formation occurs following virion maturation by the viral protease, which is supported by studies of protease-deficient viruses as discussed above [21,22]. However, rapid-harvest virus obtained from B77 ASV-infected cells contained weak gRNA dimers that dissociate under harsh extraction protocols [26]. As particle maturation continues, the B77 gRNA dimers become more stable [26]. Together, these data suggest that a weak gRNA dimer is formed at the time of particle assembly and as the virion matures, the gRNA dimer undergoes maturation as well.

The cellular location of the initial gRNA–gRNA interaction for RSV is unclear. Possible subcellular locations where gRNA dimerization can occur include: co-transcriptionally in the nucleus, in the cytoplasm, or at the plasma membrane. Data from our laboratory suggest that while RSV Gag nuclear trafficking is important for efficient gRNA packaging, nuclear trafficking alone is not sufficient for gRNA dimer formation [79]. MA domain mutations that disrupt the nuclear trafficking of RSV Gag cause a decrease in gRNA packaging while restoration of nuclear trafficking of these mutants by the insertion of a heterologous NLS rescues packaging to near wild-type levels [79]. Although gRNA packaging is restored for these mutants through the addition of an NLS, the particles produced only contain monomeric gRNA [79,80,81]. These data suggest that nuclear localization of Gag alone is not sufficient for gRNA dimerization and that the presence of a wild-type MA domain is required for gRNA dimerization. Furthermore, these data suggest that RSV Gag is capable of binding and encapsidating the gRNA as a monomer, as opposed to Moloney- murine leukemia virus (Mo-MLV) where it is thought that gRNA dimerization is a prerequisite for packaging, as determined by in vitro binding experiments [82].

For Mo-MLV, gRNA dimerization occurs co-transcriptionally in the nucleus [77,83,84]. Indeed, rapid-harvest virions obtained from Mo-MLV infected cells contain weakly-bound gRNA dimers that are sensitive to increase in temperature [85]. More mature virions harvested at longer periods following release contained more stable gRNA dimers that are less sensitive to high temperatures [85]. Furthermore, protease-deficient Mo-MLV virions contain weak gRNA dimers similar to those of rapid-harvest virions, suggesting that gRNA dimers undergo further maturation following proteolytic cleavage of the Gag protein [85]. In the case of human immunodeficiency virus type 1 (HIV-1), it is less clear where gRNA dimerization occurs, as evidence exists that suggests that HIV-1 gRNA dimerization occurs either in the cytoplasm or at the plasma membrane [86,87,88,89].

### 3.3. Where in the Cell Is gRNA Packaging Initiated?

One of the major unresolved questions in retrovirology is where in the cell does Gag select gRNA for packaging? Historically, it was thought that the Gag and the unspliced gRNA interacted at the plasma membrane where virus particle assembly occurs [5]. However, more recently, it has been observed that Gag and gRNA are present in small cytoplasmic complexes that presumably are transported to the plasma membrane where Gag multimers are added to form a complete virus particle [90,91,92]. These data suggest that the initial Gag–gRNA interaction occurs prior to plasma membrane localization.

Previously, it was thought that Gag packaged the gRNA in *cis*, meaning that Gag bound to the RNA from which it was translated [93,94]. The 5′ UTR secondary structure of the vRNA is complex, therefore it was thought the vRNA needed to be translated before Gag could bind it. This model proposed that breakdown of the RNA secondary structure by the ribosome would allow Ψ to adopt a conformation that was recognizable by Gag [93,94]. Thus, it was accepted that Gag bound the gRNA in the cytoplasm. However, the topic of exclusive *cis-*packaging in RSV was later disproven after the discovery of packaging cell lines, such as Q2bn-4D, which produce Gag proteins but cannot package their own genomes. The Q2bn-4D Gag protein is able to package heterologous RNAs in *trans* provided that they contain the Ψ sequence [60]. The *trans* packaging ability of Gag does not restrict the location of packaging to the cytoplasm; therefore, packaging could occur elsewhere in the cell, including the nucleus.

### 3.4. Impact of RSV Gag Nuclear Trafficking on gRNA Packaging

In the historical model of retroviral replication, it was thought that once Gag was translated in the cytoplasm, it traveled directly to the plasma membrane for virus assembly. However, this model was challenged when it was discovered that the RSV Gag polyprotein traffics through the nucleus [6,7,12,13,79,95]. Nuclear entry of RSV Gag is mediated by host karyopherins importin-11 and transportin-3, which interact with the NLS in MA. There is a second NLS in Gag within the NC domain, which interacts with the importin-α/β complex [7,95]. Nuclear export of Gag is mediated by interaction of the p10 NES with the host nuclear export protein CRM1 and its cofactor RanGTP [6,12,13].

Through a series of imaging and biochemical experiments, it was shown that RSV Gag nuclear trafficking is dynamic and only a small fraction of Gag present in the nucleus under steady-state conditions [6,96], RSV Gag accumulates in the nucleus upon treatment of RSV-infected cells or cells transiently expressing Gag with the CRM1 inhibitor leptomycin B (LMB). LMB treatment causes Gag to accumulate in the nucleoplasm, in nucleoli, and in discrete nuclear foci [6,13]. Similarly, mutation of any hydrophobic residue in the p10 NES (LTDWARVREEL) to alanine (e.g., Gag.L219A) also causes Gag to accumulate in the nucleus in discrete puncta [13]. The nuclear foci are sites of Gag–Gag interaction and their formation requires the presence of the NC domain [96]. Nuclear Gag foci have similar characteristics to host nuclear bodies. Nuclear bodies, such as paraspeckles, are built on a scaffolding protein or long non-coding RNA (lncRNA). The nuclear bodies themselves are tethered to the scaffold but the proteins in them are highly mobile. Using live-cell imaging, we found that the Gag nuclear foci also have a tethered or obstructed diffusion pattern [97].

Another characteristic of Gag nuclear foci is that their formation requires the NC domain of Gag, which also facilitates protein–protein and protein–nucleic acid interactions [96]. Deletion of the NC domain or replacing it with the cAMP response element-binding protein (CREB) Zip domain, which promotes protein–protein interactions, leads to the loss of Gag nuclear foci. It is unclear whether the nucleic acid binding ability of NC is the critical feature required for this interaction or whether another unknown characteristic of NC is essential [96].

To determine the possible role of Gag nuclear trafficking in retroviral replication, a Gag mutant was examined that contained the myristoylated Src membrane binding domain as an extension of the N terminus of MA (Myr1E) [81]. The Myr1E Gag mutant is strongly targeted to the plasma membrane and is reduced in nuclear trafficking ~10 fold compared to wild-type Gag [79,81]. The Myr1E mutant virus also exhibits a reduction in gRNA packaging at a level of 40% of the wild-type virus [79]. However, when an exogenous NLS from the nucleoplasmin protein is inserted into the mutant MA, Myr1E.NLS Gag is more efficiently routed into the nucleus, and gRNA packaging is restored to 80% of wild-type levels [6,79]. These studies suggested that nuclear trafficking of Gag is involved in packaging gRNA. In support of this hypothesis, in vitro biochemical studies demonstrated that the addition of nucleic acids greatly enhanced the efficiency of CRM1–Gag binding [95]. This experiment also demonstrated that Gag directly interacts with a complex consisting of CRM1, RanGTP, and RNA, defining the export complex that drives cytoplasmic localization of the viral RNP. These data supported the development of a model whereby newly synthesized RSV Gag adopts a conformation in which the NLSs in MA and NC are available for binding to import factors Transportin-3 (TNP03), importin-11, and the importin alpha-beta complex. Upon entering the nucleus, Gag sheds the importins, and the NC nucleic acid binding domain is available to interact with the vRNA. Gag–RNA binding may induce a conformational change in the protein, exposing the p10 NES that binds the CRM1–RanGTP export complex, and the retroviral RNP is exported from the nucleus [95].

Together, these data suggest that Gag nuclear trafficking is required for optimal gRNA packaging. One possibility is that Gag enters the nucleus, selects unspliced viral RNA for encapsidation, and then returns to the cytoplasm for transport to the plasma membrane. At the membrane, Gag multimers may join the viral RNP, which nucleates the assembly of the virion. There are other possible roles for Gag in the nucleus, including inhibition of vRNA splicing to favor packaging, alteration of the global host transcriptome to facilitate virus replication, or recruitment of host nuclear factors to promote virus assembly. Further studies will be needed to explore the functional relevance of nuclear localization of the RSV Gag protein.

### 3.5. Determining the Cytoplasmic Fates of Unspliced Retroviral RNA

**Figure 2:**
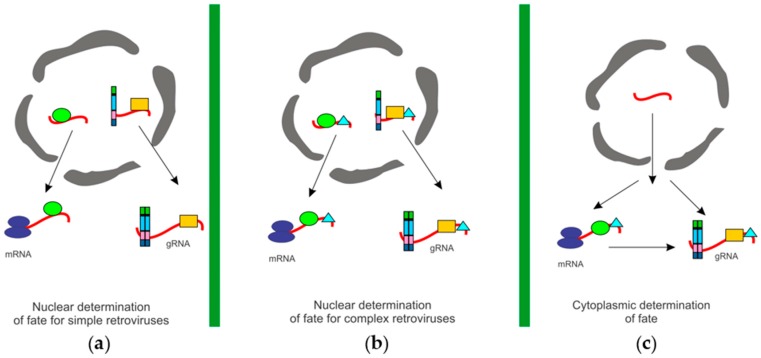
Models for unspliced retroviral RNA utilization. (**a**) Nuclear determination of vRNA fate for simple retroviruses: The cytoplasmic utilization of unspliced vRNAs may be determined by the co-transcriptional binding of specific factors that “mark” the unspliced vRNA for either packaging or translation, and the vRNAs are not interchangeable. The mechanism by which the unspliced RNA is exported from the nucleus could determine the cytoplasmic utilization of the unspliced retroviral RNA. Models include: (i) Nuclear Gag binds to the vRNA to mark it for packaging; (ii) Nuclear Gag and a host factor bind to the vRNA to mark it for packaging; or (iii) Nuclear host factors bind to the unspliced vRNA to determine its fate; (**b**) Nuclear determination of fate for complex retroviruses: Complex retroviruses encode accessory proteins that facilitate the nuclear export of unspliced or incompletely spliced retroviral RNA, such as HIV-1 Rev. In addition to Rev-like proteins, other host and viral factors also bind to the unspliced vRNA co-transcriptionally and may contribute to the fate of unspliced vRNA. Models include: (i) Nuclear viral export factor and Gag bind to the vRNA to mark it as a genome for packaging; (ii) Nuclear viral export factor, Gag, and a host factor bind to the vRNA to sort it for packaging; or (iii) Nuclear viral export factor and specific host factors bind to the unspliced vRNA to determine its cytoplasmic fate; (**c**) Cytoplasmic determination of fate: The fate of unspliced vRNAs may be defined in the cytoplasm. An unspliced vRNA can be used for either translation or packaging. A previously translated RNA can subsequently be packaged. In this scenario, host and viral factors may be bound to the unspliced vRNA in the nucleus, but their presence does not determine cytoplasmic utilization of the vRNA.

Retroviruses depend on the unspliced vRNA for dual roles: to serve as the mRNA for translation of Gag and Gag–Pol, and as the gRNA for encapsidation into virions. Presumably, these RNAs are biochemically identical, therefore the mechanism whereby retroviruses distinguish between the RNA used for translation versus the RNA used for packaging remains a mystery. Two models have been proposed as potential explanations for how retroviruses distinguish between the two unspliced vRNAs for use as gRNA or mRNA. There may be separate pools of unspliced vRNA, one for translation and the other for packaging, or a single population of vRNA may be used interchangeably, as either gRNA or mRNA (Figure 2). If the “two pools” hypothesis is correct, then the question arises as to how, when, and where the vRNAs are sorted into separate pools? One possibility is that a single metabolic pool of unspliced vRNAs exists, and the RNAs are “marked,” by viral or cellular proteins, directing them to either the translation or packaging pathways. Alternatively, two separate pools of vRNA could be physically segregated into distinct populations in the cell. The “one pool” model can be explained by two different scenarios. A vRNA can serve as either mRNA or gRNA, without translation being a prerequisite for packaging, or translation of the vRNA may be required prior to packaging [98,99]. Different retroviruses may adopt mechanisms represented by each of the models.

Murine leukemia virus (MLV) segregates its unspliced vRNA into two distinct pools that have different half-lives [100]. Inhibition of transcription in MLV-infected cells using actinomycin D for 4 h led to the production of virions deficient in gRNA, yet the virus particles were otherwise morphologically indistinguishable from particles released from untreated cells [101]. After 6–8 h of drug treatment, viral Gag and Gag–Pol proteins continued to be synthesized, reflecting the stability of the mRNA pool [101]. These observations indicate that MLV gRNA has a shorter half-life than unspliced viral mRNA, therefore gRNA and mRNA exist in two distinct populations. One explanation for these findings is that the fates of unspliced MLV RNAs are determined in the nucleus; therefore, when transcription was inhibited, new RNAs were not available to be sorted, and gRNA packaging was decreased. However, unspliced MLV mRNAs in the cytoplasm were already sorted into the translation pool; these mRNAs may be more stable so they continued to be used for translation [101].

Sorting of unspliced MLV RNAs for packaging could occur in the nucleus, especially considering that MLV gRNA dimerization occurs co-transcriptionally [77,83,84]. Because formation of the MLV gRNA dimer structure facilitates recognition by Gag, [82], the unspliced vRNA pools may be distinguished by dimerization. In other words, MLV forms dimeric gRNA in the nucleus, marking the RNA as a genome, which undergoes relatively rapid turnover. However, MLV unspliced vRNAs that fail to dimerize are transported into the cytoplasm as stable monomers, which become mRNAs destined for translation on polysomes. Identification of the host or vRNAs selectively bound to dimeric or monomeric viral RNPs would be informative to understand the vRNA sorting process mechanistically.

By contrast, experimental evidence suggests that the complex retroviruses HIV-1 and HIV-2 produce a single pool of unspliced vRNA in infected cells [98,102,103,104,105]. Actinomycin D treatment of cells infected with HIV-1 and HIV-2 led to an equivalent decrease in virion-associated gRNA and cytoplasmic unspliced vRNA, indicating that these RNAs have similar half-lives and therefore are likely derived from one pool of vRNA [102]. In the case of HIV-1, when infected cells are treated with cycloheximide to inhibit translation, packaging efficiency increases by 80%–90%, shifting the equilibrium toward the availability of gRNA used for encapsidation [105]. This observation is characteristic of a single pool in which vRNAs for translation and packaging are used interchangeably [98,105].

In an effort to identify how HIV-1 determines which vRNAs from this single pool are destined for translation or packaging, studies using HIV Gag and a luciferase reporter construct driven by the HIV-1 5′ UTR were conducted [104]. These experiments showed that as the level of Gag added in *trans* increases, the amount of reporter protein decreases, suggesting that HIV-1 Gag modulates its own translation. Furthermore, translation is increased from RNAs lacking the Ψ packaging sequence, suggesting that the regulation of protein levels is dependent upon the interaction of HIV-1 Gag with the vRNA or alternatively, deletion of Ψ from the vRNA may influence translation efficiency.

HIV-1 and HIV-2 appear to differ in their mechanism of genome encapsidation. HIV-1 Gag can package an RNA from which it was not translated (i.e., *trans* packaging). HIV-1 Gag provided in *trans* can encapsidate both HIV-1 and HIV-2 RNAs. HIV-2 Gag provided in *trans*, on the other hand, is unable to package either RNA, suggesting that HIV-2 packages the RNA from which it is translated (i.e., *cis* packaging) [103]. HIV-2 packaging is thought to occur in *cis* because Ψ is present on both the spliced and unspliced vRNA and *cis* packaging would reduce the packaging of spliced transcripts. Alternatively, it is feasible that factors in addition to Ψ, such as differences in the structure of the 5′ UTR on spliced versus unspliced RNA, subcellular localization of HIV-2 RNA unspliced and spliced vRNA populations, or the complement of host factors bound to HIV-1 RNA influence selective packaging of unspliced gRNAs rather than spliced mRNAs.

For RSV, it is currently not well understood whether there are separate pools of unspliced vRNA. One of the first experiments conducted to determine whether unspliced RSV is sorted into one or two pools compared the stabilities of the unspliced viral gRNA and the unspliced viral mRNA [106]. To determine the stabilities of the gRNA and unspliced mRNA, RSV-infected cells were treated with actinomycin D and the RNA was metabolically labeled with radioactive uridine. The results of this experiment showed that although virion production decreases faster than Gag protein synthesis, the decay rate between the two RNAs (gRNA versus mRNA) was not significantly different (6 h versus 7.5 h, respectively). These data indicate that there is one metabolic pool but it does not rule out the possibility that gRNA and mRNA are segregated by a different mechanism. Furthermore, these data supported the conclusion that prior translation of the unspliced RSV vRNA was not required for packaging to occur [106]. However, different results were obtained by examining whether RNAs that undergo nonsense-mediated decay (NMD) are packaged into virions [93]. NMD is a cellular process in which mRNAs that contain premature termination codons (PTC), which would result in truncated, potentially dangerous proteins if they were translated, are targeted for degradation. A PTC was introduced into the *gag* coding region prior to the authentic stop codon. The PTC-bearing vRNAs were packaged less efficiently than the control RNA, suggesting that most of the PTC vRNAs were translated and degraded while the remainder were packaged prior to translation.

Interestingly, the RSV Ψ sequence is present on both the spliced and unspliced vRNA. Therefore, *cis* packaging of the RSV vRNA could be invoked as a mechanism that allows Gag to bind to unspliced vRNA following translation of the viral mRNA. However, it is known that RSV Gag packages in *trans*, as demonstrated by the development of the Q2bn-4D packaging quail cell line [60]. The Q2bn-4D cell line contains a stably-integrated portion of the RSV provirus that produces a full-length vRNA, all of the viral proteins, and releases virus-like particles. The Q2bn-4D genome lacks portions of Ψ required for packaging, therefore the Q2bn-4D gRNA is not incorporated into virus particles. However, this cell line can package viral and heterologous RNAs that contain the Ψ packaging sequence.

It has been postulated that three small upstream open reading frames (uORFs 1, 2, 3) present in the 5′ UTR of the RSV RNA are responsible for regulating the balance between translation and packaging [63,107,108,109]. It was thought that translation of uORF3, which overlaps Ψ, was required for packaging: either the translation product acted as a cofactor for packaging, or the act of translation altered the Ψ sequence into a secondary structure required for its recognition by Gag [94]. Alternatively, it was also proposed that uORF3 translation was not required but that Gag competed with ribosomes for binding to the unspliced vRNA. To determine which of these models was correct, luciferase reporter constructs driven by an RSV leader that was either wild-type or contained the following mutations: (a) a Ψ deletion mutation; (b) a shortened RSV leader with all three uORFs deleted; or (c) insertion of a heterologous hairpin upstream of uORF3 that is known to inhibit ribosome movement on an RNA. The Ψ deletion and the shortened leader constructs had low packaging efficiency, as determined by RNase protection assay, but they had increased translation compared to wild-type RNA. The RSV construct that contained the extra hairpin had greatly decreased translation as expected, but packaging was only reduced by 25% compared to wild-type, suggesting that the translation of uORF3 is not required for packaging to occur.

To test whether RSV vRNAs could be used interchangeably as mRNA or gRNA, quail cells were transfected with an RSV reporter that contained the wild-type RSV leader driving the luciferase gene, and varying amounts of plasmid DNA encoding Gag were added to increase the Gag:reporter RNA ratio [94]. As the Gag:reporter ratio increased, luciferase translation decreased, suggesting that RSV Gag regulates the level of its own translation. It is possible that RSV-infected cells need to maintain an optimal level of Gag expression to limit cellular toxicity. Furthermore, as Gag levels increase, there is more Gag present to bind to vRNA for packaging, so the equilibrium is shifted toward vRNA being used as gRNA rather than as an mRNA. These data support the conclusion that there is a single metabolic pool of unspliced RSV vRNA. If a single metabolic pool of unspliced RSV RNA exists, is there a mechanism by which RSV Gag distinguishes between unspliced mRNA and gRNA? One possibility is that there is a temporal separation of functions for the vRNA. In this scenario, when the vRNA is initially transcribed following integration of the provirus, full-length Gag is not yet present in the cell so RNAs cannot be packaged; Gag must be synthesized first. Once enough Gag is made, then selection of unspliced vRNA for encapsidation ensues. When a steady-state level is reached, there is a balance between translation and packaging of vRNA.

### 3.6. Role of vRNA Structure in Selection of gRNA for Packaging

There is compelling evidence that retroviral vRNA structure plays an important role in regulating whether a vRNA molecule is used for translation or packaging. The 5′ UTR of the RSV RNA is highly structured and contains *cis*-acting signals used for competing functions such as the PBS (primer binding site), the Ψ packaging signal, the dimerization initiation signal (DIS), and the translation initiation codon (*gag* AUG). It is feasible that the higher order structure of the leader is different in spliced versus unspliced vRNA, with the unspliced leader in a favorable conformation to associate with Gag. Long-range interactions in the vRNA may be important to distinguish spliced from unspliced vRNA. Dimerization could also be more favorable in unspliced vRNA, leading to selective packaging by Gag. Furthermore, differential deposition of host factors on vRNA co-transcriptionally—for example, splicing factors and exon junction complex—would ensure that spliced vRNA is not packaged efficiently.

Similar to RSV, the HIV-1 5′ UTR is highly structured and contains multiple stem loops including the trans-activation response element (TAR), polyA loop, PBS, DIS, Ψ, and the *gag* initiation codon that are required for transcriptional control, reverse transcription, dimerization, packaging, and translation. Viruses containing mutations in the 5′ UTR deleting the PBS, polyA, and TAR binding loop but maintaining the structure of the 5′ UTR are competent for packaging with only a minor decrease compared to wild-type [110,111]. However, deletion of regions downstream of the PBS leads to a decrease in packaging and transduction of lentiviral vector constructs [112], suggesting that this region of the HIV-1 RNA is a minimal site required for gRNA packaging. In vitro analysis of HIV-1 vRNA revealed that long range interactions occur between the 5′ UTR and the *gag* coding region are proposed to promote packaging, dimerization, and regulate splicing [113,114]. It has been proposed that the long range interactions form an RNA conformation that must undergo a riboswitch to a structure that favors packaging and dimerization [115]. While it was previously thought that this riboswitch did not have an effect on translation of the unspliced viral mRNA [116], NMR analysis of the HIV-1 5′ UTR suggests that the vRNA may adopt different structures to promote packaging or translation [117,118]. When the HIV DIS is base-paired with a complementary region in U5, the Gag AUG is unpaired, and the structure is proposed to promote translation [117,118]. If the Gag AUG is base-paired with a complementary region in U5, Ψ and the DIS are exposed, thus anticipated to promote packaging and genome dimerization [117,118].

The 5′ UTRs of other retroviruses also adopt long range interactions. The exact location of the feline immunodeficiency virus (FIV) packaging signal is still unclear. It is suspected that the FIV vRNA contains two packaging signals, a 150-nucleotide region upstream of the splice donor that is present on both the spliced and unspliced vRNAs, and one near the 5′ end of the *gag* coding region [119,120,121,122,123,124,125]. In vitro and in vivo studies revealed that a long range interaction between nucleotides in RU5 and a palindromic sequence in the *gag* coding region is essential for efficient genome encapsidation [125,126]. Mason-Pfizer monkey virus (MPMV) RNA also contains a bipartite packaging signal located in the initial 50 nucleotides and the last 23 nucleotides of the 5′ UTR extending through the first 120 nucleotides of *gag* [127,128,129,130,131]. Selective 2'-hydroxyl acylation analyzed by primer extension (SHAPE) and mutational analysis of the MPMV 5′ UTR revealed possible long range interactions between U5 and the *gag* coding region that may be required for genome dimerization and packaging [132,133]. Similarly, the mouse mammary tumor virus (MMTV) packaging signal is thought to span from repeat (R) through the first 120 nucleotides of *gag*, and the MMTV U5 sequence engages in long range interactions with the *gag* coding region to influence genome dimerization and packaging [134,135,136,137]. If the RSV leader sequence also adopts different conformations, it is possible that RSV Gag binds preferentially to a gRNA with a specific 5′ UTR structure.

### 3.7. Contribution of Host Factors to vRNA Sorting

Yet another possibility is that the RNAs are “marked” by host factors that sort the unspliced RNAs either for packaging or translation shortly after they are transcribed in the nucleus. It has been proposed that proteins that bind to an RNA in the nucleus determine its cytoplasmic fate [1,2,3] (Figure 2). For instance, zipcode-binding protein-2 (ZBP2) binds to β-actin mRNA in the nucleus to mark it for localization to the leading edge of fibroblasts, where actin is usually very active [138]. Therefore, it is intriguing to propose that different sets of host and viral factors in the nucleus may be responsible for marking and sorting RSV unspliced vRNAs to be used for packaging or translation.

To identify cellular factors that potentially play a role in marking retroviral RNAs for packaging or translation, affinity pulldown experiments were performed on cell lysates using the 5′ UTR sequences of HIV-1, RSV, and spleen necrosis virus as bait, and interacting host proteins were identified by mass spectrometry [139]. This set of host nuclear proteins included factors that were shared among the examined retroviruses, as well as those that were distinct for each. Cellular factors identified were involved in transcription and mRNA processing, nucleocytoplasmic transport, translation and ribosome biogenesis, noncoding RNA biology, and intracellular transport. Domain analysis of factors that interacted with the 5′ UTRs included nucleotide binding factors, DNA/RNA helicases, double-stranded RNA binding proteins, heterogeneous nuclear ribonucleoproteins (hnRNPs), splicing factors, ribosomal proteins, scaffold proteins and proteins involved in the DNA damage response. Although many of these factors had been shown to function in retrovirus replication, others were novel and bear further examination. In a proteomic analysis of human proteins that bind to HIV-1 RNA, 32 proteins were identified. In that study, Matrin 3 (MATR3), a component of nuclear matrix, was found to be essential for Rev-mediated nuclear export of unspliced and partially-spliced HIV-1 RNA [140]. In addition, cellular RNAs such as long non-coding RNAs (lncRNAs) may also be involved in determining the fate of the unspliced vRNAs. For example, nuclear paraspeckle assembly transcript 1 (NEAT1) lncRNA, which nucleates the formation of nuclear paraspeckles, modulates HIV-1 replication [141,142]. Thus, the interplay of host proteins and noncoding RNAs undoubtedly contributes to marking and sorting retroviral unspliced RNA for packaging or translation (Figure 2), although the molecular mechanisms underlying this process are still under active investigation.

### 3.8. Unspliced Retroviral RNA Nuclear Export

After RNA processing, the RSV unspliced RNA must leave the nucleus. Nuclear export of cellular RNAs is usually linked to splicing [143]. When an intron is spliced from an RNA, the two exons are ligated to form an exon–exon junction. The exon junction complex (EJC) is a family of proteins that recognizes and binds the exon–exon junction. Then the EJC member Aly/REF recruits the Tap/ nuclear transport factor 2 like export factor 1 (NXT1) nuclear export complex and the RNA is exported from the nucleus [144]. Incorrectly processed mRNAs are retained in the nucleus by the translocated promoter region protein (TPR) nuclear protein to prevent translation of an aberrant protein. TPR binds to the 5′ splice site of unspliced RNAs and tethers them to the nuclear pore complex (NPC) [144,145,146]. The N-terminus of TPR contains a coil-coil domain that tethers TPR to the NPC [147,148,149]. The C-terminus of TPR contains domains for interactions with CRM1, importin α, and importin β [150,151]. TPR then recruits exosomal proteins that degrade the questionable RNA [144].

Retroviruses must circumvent these quality control mechanisms set in place by the cell. Complex retroviruses encode accessory proteins that facilitate nuclear export of unspliced retroviral RNA [152]. HIV-1 encodes the Rev protein which binds to the Rev response element (RRE) in the 3′ end of unspliced vRNA to facilitate its export through the CRM1 pathway [152]. A variety of cellular factors have been identified that contribute to Rev-mediated vRNA export, including MATR3, DEAD-box helicase 3 (DDX3), splicing factor proline-glutamine rich (SPFQ), and regulator of nonsense transcripts 1 (UPF1) [140,153,154,155]. However, simple retroviruses including MPMV and RSV do not encode Rev-like proteins, thus they co-opt host factors such as Tap/NXT1 to export the unspliced vRNA from the nucleus [156,157,158,159]. Tap/NXT1 facilitates the nuclear export of unspliced retroviral RNA by binding to *cis*-acting sequences in the vRNA. For MPMV, Tap binds to the constitutive transport element (CTE) [156,157,158,159]. For RSV, it is thought that Tap indirectly interacts with the direct repeat (DR) sequences, which flank the *src* coding region in the unspliced vRNA [160].

To identify the RSV RNA elements and the host factors required for nuclear export of the unspliced vRNA, dominant-negative mutants and reporter constructs containing one or two of the RSV DR sequences or the RSV Ψ sequence were created [160]. Nuclear export of the reporter RNAs was measured by chloramphenicol acetyltransferase (CAT) activity in cell lysates, indicating that the reporter mRNA was used as a template for translation in the cytoplasm [160]. Transfection of cells with the DR-containing constructs led to CAT activity, but CAT expression was not detected in cells containing the Ψ-containing constructs, even in the presence of Gag. The authors concluded that the cellular host factors Tap and Dbp5, an RNA helicase located in the NPC, interact with DRs of RSV to facilitate the export of unspliced vRNA in a CRM1- and Gag-independent manner [160,161]. However, because the output of this assay was translation of CAT mRNA, the data do not provide any information about the role of Gag, DR elements, Tap, or Dbp5 in gRNA packaging.

To determine the effect of Dbp5 mutation on the cellular localization of unspliced RSV RNA, a wild-type RSV proviral plasmid and a wild-type or dominant-negative Dbp5 mutant were transfected into cells [160]. The cellular localization of the unspliced RSV RNA was determined via fluorescence in situ hybridization (FISH) using a probe against the *gag* coding region. Although unspliced RSV RNA was retained in the nucleus in the presence of the Dbp5 mutant, a portion did localize to the cytoplasm, although the effect on gRNA packaging was not assessed in this study [160]. Therefore, it remains unknown whether Tap/Dbp5 facilitates export of RSV gRNA. It remains possible that whereas Tap/Dbp5 facilitates the export of the unspliced RSV vRNA for translation, a different pathway promotes export of the unspliced vRNA for packaging (Figure 2).

### 3.9. Nuclear Trafficking of Other Retroviral Gag Proteins

**Figure 3:**
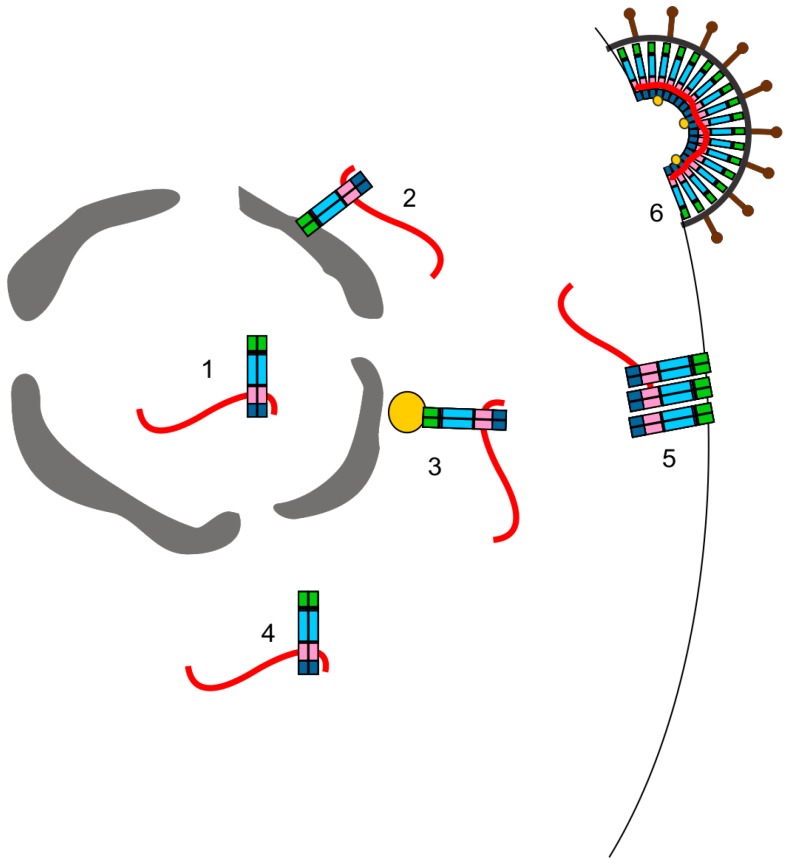
Potential subcellular sites of the initial Gag–gRNA interaction. Gag and the vRNA may first interact: (1) in the nucleus; (2) along the cytoplasmic face of the nuclear envelope; (3) at the microtubule organizing center (MTOC; yellow); (4) in the cytoplasm; or (5) at the plasma membrane. In each case, the viral ribonucleoprotein complex binds to the plasma membrane and interacts with other Gag proteins to form a virion that (6) buds from the plasma membrane.

The discovery that RSV Gag nuclear trafficking played a role in efficient gRNA encapsidation is novel to the field [79] (Figure 3). Since then, advances in microscopy and other methods have allowed other researchers to observe nuclear trafficking events of other retroviral and retrotransposon Gag proteins (reviewed in [162]). Previously, the Gag protein of the simple retrovirus MLV was detected within the nucleus of infected NIH3T3 cells [163]. Subcellular fractionations and immunoelectron microscopy staining for MLV Gag revealed that approximately 18% of the Gag protein was present within the nucleus of infected cells. It was proposed that nuclear MLV Gag could play a role in packaging the genomic RNA, but no experiments were done to test that idea.

In contrast to RSV, MLV Gag is myristoylated at the N-terminus. A mutant MLV Gag lacking the myristic acid addition site has increased nuclear targeting (32%), suggesting that myristoylation of MLV Gag regulates nuclear trafficking of MLV Gag [163]. Furthermore, temperature sensitive MLV Gag mutants lacking various regions of CA failed to enter the nucleus, suggesting the presence of an NLS within the capsid domain [163]. Additionally, the NC domain of MLV Gag has been observed to be present within nucleoli, although it is not known whether this is important for early replication events (pre-integration complex (PIC) nuclear entry and provirus integration) or late events (assembly) [19]. Furthermore, it is unclear whether full-length MLV Gag is present in nucleoli [19]. The role of MLV Gag nuclear trafficking is unknown, but recent results suggest that nuclear MLV Gag plays a role in gRNA dimerization and the encapsidation of non-viral RNAs into virions [reviewed in [162]].

Nuclear localization of the HIV-1 Gag protein was initially observed in insect cells, and a putative NLS was mapped to the CA domain [164]. In human cells, it was observed that HIV-1 Gag and the vRNA co-localize at the perinuclear microtubule organizing center (MTOC) and that this may be the initial interaction site of HIV-1 Gag and gRNA [165] (Figure 3). Subsequently, it was reported that the HIV-1 Gag-green fluorescent protein (GFP) fusion protein expressed from a doxycycline-inducible provirus localizes within nucleoli of HeLa cells [19]. When expressed alone, the HIV-1 NC domain traffics to nucleoli, and two independent nucleolar localization signals were mapped within NC [19]. An NLS in NC was recently identified in the linker region between the Cys-His boxes [166]. The HIV-1 NC domain has previously been observed within the nucleus during early infection [167], but whether the nuclear targeting activity of the NC domain plays a role in directing Gag to the nucleus during packaging or virus assembly is unknown. It will be important to investigate whether HIV-1 Gag nuclear trafficking has a biological function in virus replication. However, the study of HIV-1 Gag nuclear trafficking will be challenging because Gag does not accumulate to high levels in the nucleus with LMB treatment, suggesting that the protein does not contain an intrinsic CRM1-dependent nuclear export signal [168,169,170].

The Gag protein of FIV, a lentivirus that infects cats, also traffics to the nucleus [168]. FIV Gag can be observed within the nucleus under steady-state conditions and like RSV, is exported from the nucleus by CRM1. LMB treatment of cells expressing FIV Gag, either transiently transfected or in the context of an infection, causes Gag to accumulate in the nucleus and nucleoli in an NC-dependent manner [168]. Furthermore, FIV Gag and gRNA co-localize at the cytoplasmic face of the nuclear envelope in HeLa cells [171] (Figure 3). Deletion of the FIV Ψ packaging sequence prevents Gag from co-localizing with the gRNA at the nuclear envelope and instead Gag is present within the cytoplasm while FIV gRNA remains near the nuclear envelope. These data suggest that the initial Gag–gRNA interaction for FIV may occur at the cytoplasmic face of the nuclear envelope, although more studies are needed to explore this possibility.

Human prototype foamy virus (PFV) has several features that distinguish it from the other orthoretroviruses. For instance, the PFV Gag protein is not processed and the Gag–Pol protein is only partially processed, as integrase is cleaved from Pol during maturation (reviewed in [172]). PFV Gag is strongly localized to the nucleus of infected cells [173]. During prophase, PFV Gag accumulates in nuclear foci but it is tethered to mitotic chromatin via its interaction with histones H2A and H2B to facilitate integration of the proviral DNA [174,175]. It has also been reported that PFV Gag may be exported from the nucleus via CRM1 [175,176]. Although PFV nuclear trafficking is important for early events of replication, it remains to be seen whether nuclear localization is required for assembly.

In addition to retroviruses, the Gag proteins of retroelements such as the yeast LTR retrotransposon Ty1 and the Tf1 retrotransposon are associated with the nucleus [177,178]. Although Tf1 Gag is present in the nucleus under steady-state conditions [178], Ty1 Gag nuclear trafficking is transient, making it difficult to detect under standard conditions [177]. However, when Ty1 Gag is expressed in a temperature sensitive mutant strain of *mex67* (the yeast ortholog of the human Tap RNA nuclear export protein), Gag accumulates at the nuclear envelope [177]. In the absence of Gag, the Ty1 vRNA is trapped and degraded in the nucleus, suggesting a role for Gag in RNA nuclear export and/or stabilization of Ty1 RNA [177].

Together, these data provide evidence for the role of Gag nuclear trafficking in the replication of several retroviruses and retroelements. Although the function of Gag nuclear trafficking is unclear for many viruses, it appears that there may be a link with either selection of gRNA for packaging or with vRNA stability. These possible functions for nuclear Gag proteins are consistent with the major roles of Gag to select and bind the unspliced vRNA, “marking” it as genome for encapsidation into virions. Whether the nucleus is the site of the initial Gag–gRNA interaction that leads to genome packaging is an intriguing possibility that is currently under study.

### 3.10. Conclusions and Remaining Questions

The encapsidation of gRNA is required for the production of infectious virions, yet many fundamental questions concerning vRNA sorting, gRNA packaging, and genome dimerization remain unclear. For example, how do retroviruses distinguish unspliced mRNA from gRNA? It is likely that retroviruses take advantage of host proteins and noncoding RNAs that interact with newly synthesized vRNA in the nucleus to direct unspliced vRNA toward the splicing, mRNA export, or encapsidation pathway. Where in the cell does the initial Gag–gRNA interaction occur? Intracellular sites for Gag–vRNA interaction that have been suggested by experimentation include within the nucleus, at the cytoplasmic face of the nuclear envelope, at the MTOC, at specialized sites in the cytoplasm, and at the plasma membrane (Figure 3). It is not clear whether each of these subcellular locations is the sole place where Gag selects and binds gRNA, or whether multiple interaction sites are possible. It is also feasible that different retroviral Gag proteins have adapted to their specialized intracellular environments and therefore use different strategies to identify, select, and bind gRNA.

When and where does gRNA dimerization occur and is vRNA dimer formation a prerequisite for packaging? Again, the data suggest that retroviruses may develop different strategies for genome dimerization. MLV appears to be at one end of the spectrum, with evidence for co-transcriptional dimer formation and recognition of gRNA dimers by Gag, whereas the HIV-1 and RSV Gag proteins appear to select monomeric genomes that later dimerize in the cytoplasm, at the plasma membrane, or in virions. Because dimerization of the genome is absolutely required for replication, retroviruses may stack the deck by creating opportunities for gRNA to dimerize at multiple steps along the pathway, ranging from the initial selection of gRNA to the release of nascent virions. This requirement for genome dimerization and packaging makes these processes important targets for the development of novel treatment against retroviral infection and for the optimization of therapeutic retroviral vectors to correct genetic defects. Finally, the recent discovery that the Gag proteins of several retroviruses, including RSV, HIV-1, FIV, and MLV, undergo nuclear localization may reveal previously unappreciated insights into the initial step in the selection of retroviral gRNA, ultimately leading to the encapsidation the genome into virions.
